# Spasmodic Rigidity of the Os

**Published:** 1888-11

**Authors:** 


SPASMODIC RIGIDITY OF THE OS.—This is really a state of tetanus in the anterior lip of the uterus, according to Doléris, found usually in neurotic primiparæ when premature rupture of the membranes has allowed the waters to entirely escape. The suffering is, of course, intense, and the patient's state of mind pitiable. Under chloroform the condition disappears at once, and labor, as a rule, progresses favorably. Dr. Doléris utters a protest against antispasmodics of all kinds in this complication, and against the classic views upon the subject of spasmodic rigidity that are found in books.—*Post-Graduate.*

